# Comparative Evaluation of Different Blood Collection Sites for the Assessment of Beta‐Hydroxybutyric Acid Concentrations Using a Handheld Meter in Sheep

**DOI:** 10.1002/vms3.70592

**Published:** 2025-08-26

**Authors:** Mehmet Akköse, Ufuk Kaya, Murat Onur Yazlik, Murat Göcen, Mehmet Rıfat Vural

**Affiliations:** ^1^ Department of Livestock Dalaman Agricultural Enterprise, General Directorate of Agricultural Enterprises Muğla Türkiye; ^2^ Department of Biostatistics Faculty of Veterinary Medicine Hatay Mustafa Kemal University Antakya Türkiye; ^3^ Department of Obstetrics and Gynecology Faculty of Veterinary Medicine Ankara University Ankara Türkiye; ^4^ Graduate School of Health Sciences Faculty of Veterinary Medicine Ankara University Ankara Türkiye

**Keywords:** β‐hydroxybutyric acid, FreeStyle Optium Neo H, ewe, hyperketonaemia

## Abstract

Hyperketonaemia is a common problem in periparturient sheep. This study aimed to evaluate the performance of FreeStyle Optium Neo H (FS) for measuring blood β‐hydroxybutyric acid (BHBA) concentrations in sheep. Whole‐blood BHBA concentrations were measured using the FS from the jugular and ear veins of 175 clinically healthy, late‐gestation (*n* = 56) and postparturient (*n* = 119) ewes. Jugular blood serum samples were also used for the reference sampling site. Blood BHBA concentrations measured with the FS from the jugular and ear veins showed a high positive correlation with the serum concentrations measured using the reference method (*r* = 0.84, *r* = 0.81, respectively). According to the Bland–Altman plot and limits of agreement, when compared to the reference method, FS recorded lower BHBA concentrations of 0.03 and 0.04 mmol/L from the jugular and ear veins, respectively. Passing–Bablok regression analysis revealed an intercept of −0.125 (95% CI: −0.148 to −0.078) and a slope of 1.250 (95% CI: 1.111–1.304) for the jugular vein and an intercept of −0.005 (95% CI: −0.089 to −0.018) and a slope of 1.000 (95% CI: 0.909–1.111) for the ear vein. The receiver operating characteristics (ROC) analysis determined that the optimal thresholds of the FS for the jugular and ear veins were, respectively, ≥0.8 mmol/L (sensitivity, 87.5%; specificity, 97.6%, AUC, 0.896) and ≥0.6 mmol/L (sensitivity, 87.5%; specificity, 91.0%, AUC, 0.887) for the identification of sheep with pregnancy toxaemia (PT). In conclusion, although there was constant and proportional errors indicating non‐agreement with the reference method in the Passing–Bablok regression equation, FS's diagnostic performance was good enough to identify ewes with PT or hyperketonaemia.

## Graphical abstract

Whole‐blood BHBA concentrations were measured using the FreeStyle Optium Neo H (FS) from the jugular and ear veins of 175, late‐gestation (*n* = 56) and postparturient (*n* = 119) ewes. Jugular blood serum samples BHBA concentrations were measured using the reference method (Randox). Passing–Bablok regression analysis revealed an intercept of −0.125 (95% CI: −0.148 to −0.078) and a slope of 1.250 (95% CI: 1.111–1.304) for the jugular vein and an intercept of −0.005 (95% CI: −0.089 to −0.018) and a slope of 1.000 (95% CI: 0.909–1.111) for the ear vein. The optimized thresholds of the FS for the jugular and ear veins were, respectively, ≥0.8 mmol/L (Sensitivity, 87.5%; Specificity, 97.6%) and ≥0.6 mmol/L (Sensitivity, 87.5%; Specificity, 91.0%) for the identification of sheep with hyperketonaemia. In conclusion, although there were constant and proportional errors indicating non‐agreement with the reference method in the Passing–Bablok regression equation, FS's diagnostic performance was good enough to identify ewes with PT or hyperketonaemia.

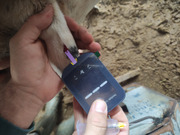

## 1 Introduction

Pregnancy toxaemia (PT, hyperketonaemia, twin‐lamb disease, or ketosis) is one of the most important metabolic diseases of small ruminants, regardless of yield orientation, often resulting in a flock problem (Silva et al. 2022). The mortality rate of affected animals is up to 80% (Rook 2000). PT incidence is the highest during the last 2 weeks of pregnancy, when the energy requirements for fetal growth are at their peak. Ewes carrying more than one foetus often suffer from PT (Silva et al. 2022).

Elevated blood β‐hydroxybutyric acid (BHBA) concentration is a diagnostic marker for PT. The laboratory measurement of blood BHBA concentration is considered the gold standard in the diagnosis of ketosis (Oetzel 2007). In healthy sheep, serum BHBA concentrations are below 0.8 mmol/L, while serum BHBA concentrations between 0.8 and 1.6 mmol/L indicate moderate hyperketonaemia, and BHBA concentrations of ≥1.6 mmol/L indicate severe hyperketonaemia (or higher risk for developing clinical ketosis) (Ji et al. 2023; Öztürk and Mamak 2023). Since laboratory measurement of BHBA is expensive and time‐consuming, several studies have assessed on‐site use of handheld devices, originally developed for monitoring ketotic diabetes in humans, for measuring blood BHBA concentrations to diagnose PT or ketosis in sheep (Pichler, Damberger, Schwendenwein, et al. 2014; Panousis et al. 2012, 2018; Tümer and Kılınç 2023; Turgut et al. 2024).

While several studies have used blood samples collected from the jugular vein to measure BHBA concentrations for diagnosing hyperketonaemia in sheep (Araújo et al. 2020; Jones et al. 2018; Ratanapob et al. 2019; Panousis et al. 2012; Tümer and Kılınç 2023; Turgut et al. 2024), only one study has evaluated BHBA concentrations using blood samples collected from the ear vein (Pichler, Damberger, Schwendenwein, et al. 2014). Regarding other species, Uztimür et al. (2025) evaluated two point‐of‐care devices for diagnosing hyperketonaomia in goats using the jugular and ear veins. The sampling point matters because blood BHBA concentrations may vary for samples from different peripheral sites of the body. Mahrt et al. (2014) reported that blood from the mammary vein contained less BHBA than blood samples from the jugular or coccygeal veins. Wilhelm et al. (2013), for example, compared BHBA concentrations in blood samples from four different sampling sites (mammary vein, jugular vein, and left and right external saphenous veins) in lactating and non‐lactating dairy cows. For lactating dairy cows, they found higher BHBA concentrations in blood samples from the jugular and saphenous veins, compared to blood from the mammary vein. However interestingly, they reported that BHBA concentrations in non‐lactating cows were lower in blood samples from the jugular vein compared to those from the mammary vein. They suggested that this was caused by the different rates of BHBA consumption in the tissues drained by these veins, especially the cerebral and mammary tissues (Wilhelm et al. 2013). In sheep, the brain's use of ketone bodies (Kammula 1976) may result in a lower concentration of ketones in blood sampled from the jugular vein than in blood from other peripheral vessels. For the regular monitoring of blood ketone concentrations in sheep, it is therefore important to compare blood BHBA concentrations using a sampling technique that is both minimally invasive and easily applicable by sheep breeders, specifically for the ear vein. Accordingly, the present study aimed to validate the FreeStyle Optium Neo H for the measurement of BHBA concentrations at two different blood sampling sites (jugular and ear veins) and to evaluate the diagnostic accuracy of the FreeStyle Optium Neo H for detecting hyperketonaemia in sheep.

## 2 Material and Method

### 2.1 Animal Material

This validation and diagnostic accuracy study was conducted with the approval of the Local Ethics Committee for Animal Experiments of Muğla Sıtkı Koçman University. This study was conducted at four sheep farms in the Serinhisar district in Denizli province and one sheep farm in Emirdağ district in Afyonkarahisar province between May 2022 and November 2023. A total of 175 late‐pregnant (Karya, *n* = 47; Konya Merino, *n* = 9) and post‐parturient (Karya, *n* = 75; Konya Merino, *n* = 10; Pırlak, 34) sheep of the Karya, Konya Merino, and Pırlak breeds were used in the study. The ewes, which were between 1 and 5 years old, were fed ∼1 kg per day of concentrated feed, consisting of a mixture of barley and wheat, and wheat straw. Fresh water was given ad libitum to all sheep.

### 2.2 Sample Collection and BHBA Measurement

BHBA concentrations were measured using blood samples taken from the jugular and ear veins of the sheep with the FreeStyle Optium Neo H device. Jugular blood samples were also used for serum BHBA measurement with an otoanalyzer using Randox reagent kits as the reference method. During sampling, the sheep were handled by their breeders, and the jugular blood samples were collected into vacuum‐packed, sterile, plain tubes. The BHBA concentrations of the jugular blood samples in the tubes were measured using the handheld device immediately after being collected. The ear vein blood samples were collected using blood collection needles. A drop of ear blood was placed on the test strip of the handheld device, and the BHBA concentration was measured within 10 s. Before use, the handheld device was calibrated with the calibrator of the strips. Sample collection and BHBA measurements at both blood sampling sites were performed in a single session by (a) collecting the jugular blood samples, (b) measuring the BHBA concentrations of the jugular blood samples collected into tubes using the handheld device, and (c) puncturing the ear vein and measuring the blood BHBA concentrations with the handheld device. The analytical range of the FreeStyle Optium Neo H for BHBA measurement was 0.0–8.0 mmol/L. The jugular blood samples were centrifuged at 3500 rpm for 10 min, and the extracted sera were stored at −20°C until laboratory analysis. All laboratory measurements of the serum samples were completed within 6 months of the blood sampling. Serum BHBA concentrations were measured with an automated biochemistry analyzer (RX Monaco, Randox Laboratories) using Randox Ranbut assay reagents as the reference method. In brief, the principle of this colorimetric analysis method is that, simultaneously with the reduction of NADH to NAD+, d‐3‐hydroxybutyrate dehydrogenase catalyzes the oxidation of BHBA to acetoacetate. This reaction results in a colour change in the samples, the absorbance of which is measured photometrically.

### 2.3 Statistical Analysis

Statistical analyses were performed using the MedCalc (Belgium) statistical software. The normality of the data distribution was determined according to the Shapiro–Wilk test. In all statistical analyses, the significance level was set as *p* < 0.05. The Spearman correlation coefficient between the BHBA concentrations measured with the reference method and the FreeStyle Optium Neo H device in samples from different vessels was calculated. The agreement between the BHBA concentrations obtained with the reference method and FreeStyle Optium Neo H was analyzed by the Bland–Altman test, in which the 95% confidence interval (CI) of agreement should include 95% of mean differences. The systematic and proportional bias between the BHBA concentrations measured by the reference method and with the FreeStyle Optium Neo H was analyzed using Passing–Bablok regression analysis. The resulting slope and intercept were evaluated by determining whether the 95% CIs included 1 and 0, respectively. Accordingly, assumptions were made based on a perfect agreement between the two methods being represented by an intercept (a) of 0 and a slope (b) of 1. It was assumed that the CI of the intercept including 0 would indicate no considerable constant bias, and not including 0 would indicate that the two tested methods differed at least by a constant concentration of BHBA. For the slope, it was assumed that 1 being included in the CI would indicate the absence of bias, and 1 not being included would indicate the presence of at least a proportional difference between the two methods. Inter‐rater agreement (Cohen's kappa coefficient) was determined to demonstrate the agreement of the FreeStyle Optium Neo H device with the reference method in detecting hyperketonaemia in blood from the different blood sampling sites. The kappa coefficients were classified as indicating none (0.0–0.20), minimal (0.21–0.39), weak (0.40–0.59), moderate (0.60–0.79), strong (0.8–0.90), or almost perfect (>0.90) agreement (McHugh et al. 2012).

The intra‐assay coefficient of variations (CVs) was calculated from five consecutive measurements of whole‐blood samples with FreeStyle Optium Neo H and serum samples with reference method (Randox). The % CV for each sample is calculated by finding the standard deviation of results, dividing that by the duplicate mean, and multiplying by 100. The average of the individual CVs is reported as the intra‐assay CV (Riis et al. 2021).

Based on the serum BHBA concentrations measured by the reference method, the sheep were classified as healthy (<0.8 mmol/L) and hyperketonaemic (≥0.8 mmol/L). A receiver operating characteristics (ROC) analysis was conducted to optimize the thresholds of the FreeStyle Optium Neo H device in detecting hyperketonaemia at the different blood sampling sites. The optimum thresholds were determined according to the Youden index, calculated on the basis of the combination of the highest sensitivity and specificity. The areas under the ROC curves (AUC) were used to determine the ability of FreeStyle Optium Neo H to discriminate between healthy and hyperketonaemic sheep. Interpretation of the intermediate AUC values was defined as low (0.5< AUC ≤0.7), moderate (0.7< AUC ≤0.9), or high (0.9< AUC ≤1) accuracy (Gardner and Greiner 2006).

## 3 Results

**FIGURE 1:**
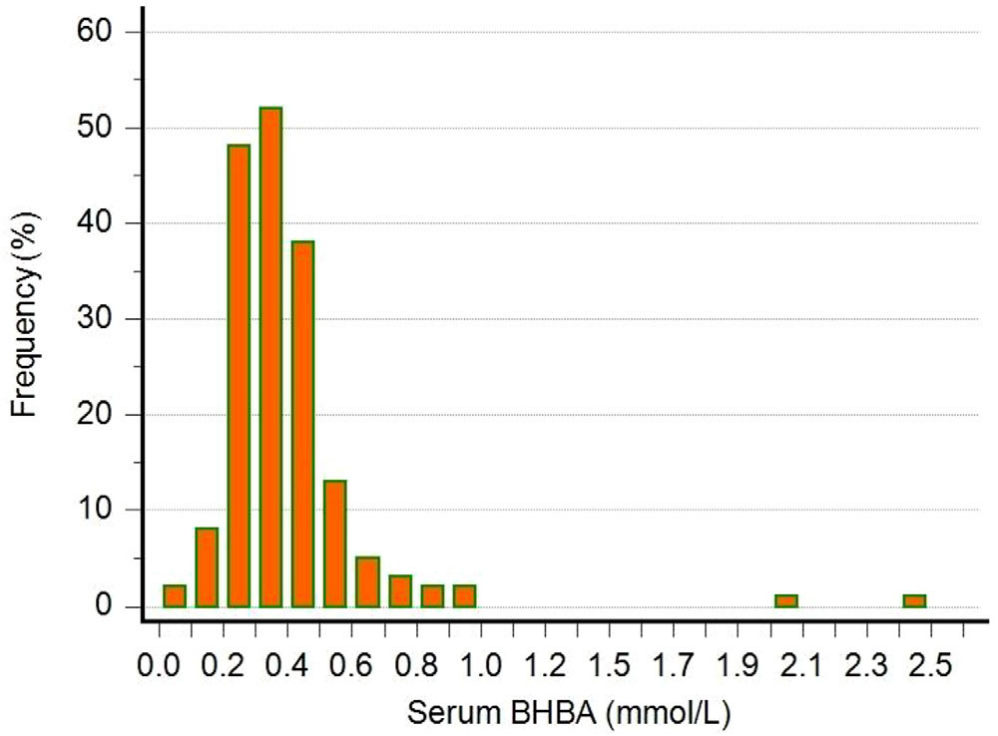
Distribution of serum BHBA concentrations measured with the reference method (Randox). Samples were collected from late gestation and postparturient ewes (*n* = 175) in the Aegean and Central Anatolian regions of Türkiye.

Table 1 shows the descriptive statistics of the jugular BHBA concentrations measured with the Randox kit and the jugular and ear BHBA concentrations measured with the FreeStyle Optium Neo H device. The BHBA concentrations measured with the Randox kit ranged between 0.0 and 2.46 mmol/L, whereas the BHBA concentrations measured with FreeStyle Optium Neo H ranged between 0.0 and 3.1 mmol/L and 0.1 and 2.9 mmol/L in blood sampled from the jugular and ear veins, respectively. Figure 1 presents the distribution of the serum BHBA measurements using the reference method (Randox). The intra‐assay coefficients of the FreeStyle Optium Neo H and reference method (Randox) were calculated as 0%. The mean, the standard deviation, and raw data of all measurements are presented in Table S1.

**TABLE 1:** Descriptive statistics of the serum BHBA concentrations in sheep measured by the reference method (Randox) and whole‐blood BHBA concentrations measured with the FreeStyle Optium Neo H device in samples from jugular and ear vein.

Sample method and site	*n*	Minimum	Maximum	Mean	Std. deviation	Median	Interquartile range
Randox jugular BHBA (mmol/L)	175	0.00	2.46	0.41	0.25	0.37	0.17
FS jugular BHBA (mmol/L)	175	0.00	3.10	0.38	0.33	0.30	0.20
FS ear BHBA (mmol/L)	175	0.10	2.90	0.37	0.30	0.30	0.20

Abbreviations: BHBA, β‐hydroxybutyric acid; FS, FreeStyle Optium Neo H.

The whole‐blood BHBA concentrations measured with the FreeStyle Optium Neo H in the jugular and ear veins were highly correlated with the serum BHBA concentrations measured with the Randox kit (*r* = 0.84 [95% CI: 0.79–0.88] and *r* = 0.81 [95% CI: 0.75 to 0.86], respectively).

**FIGURE 2:**
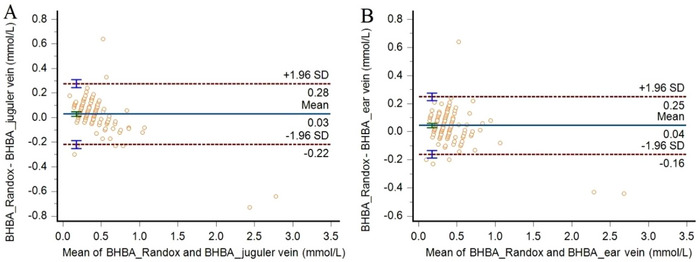
Bland–Altman plot. Differences in whole‐blood BHBA concentrations were measured with FreeStyle Optium Neo H for samples from the jugular (A) and ear (B) veins, and the serum BHBA concentrations were calculated with the reference method (Randox). Samples (*n* = 175) were collected from late gestation and postparturient ewes in the Aegean and Central Anatolian regions of Türkiye.

Bland–Altman plots were used to analyze the level of agreement between the BHBA concentrations measured with the two methods and for samples from the two blood collection sites (Figure 2). According to the Bland–Altman plots and limits of agreement, the BHBA concentrations measured using the FreeStyle Optium Neo H device for samples from the jugular and ear veins were, respectively, 0.03 mmol/L (95% CI: 0.01–0.05) and 0.04 mmol/L (95% CI: 0.03–0.06) lower than those detected with the reference method.

**FIGURE 3:**
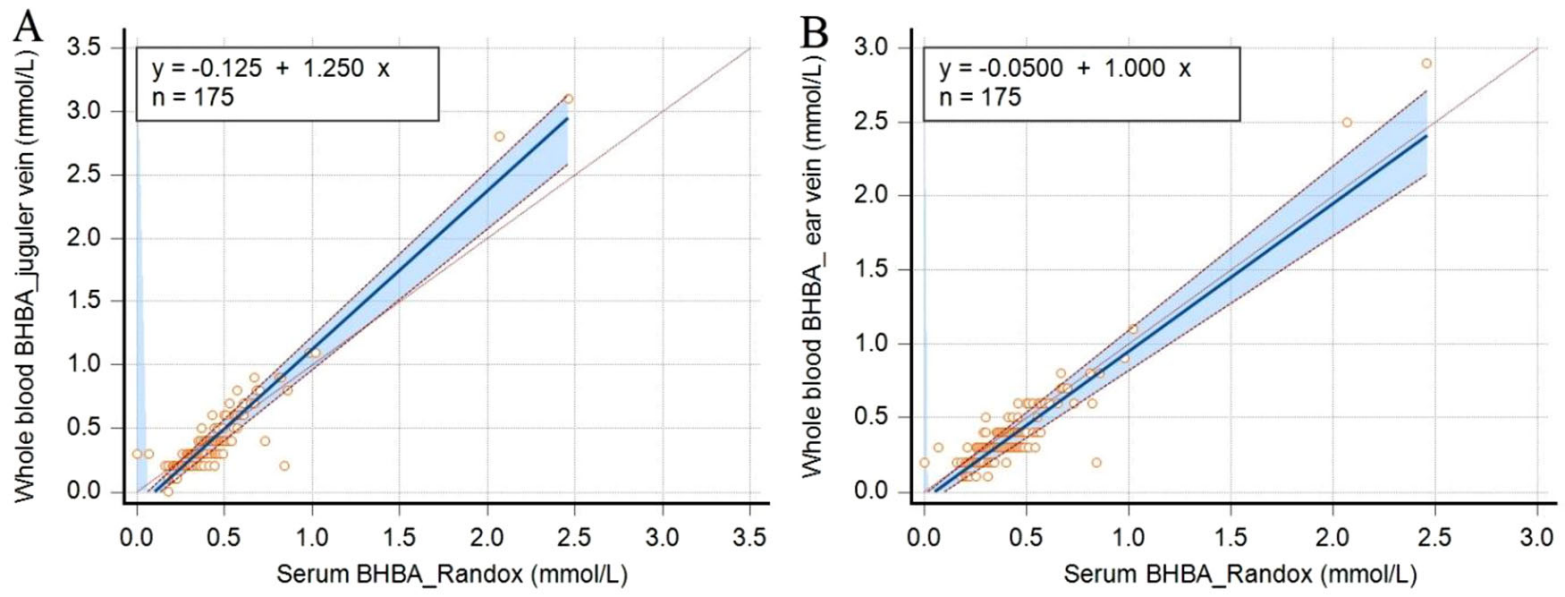
Passing–Bablok regression equation between whole‐blood beta‐hydroxybutyric acid (BHBA, mmol/L) measured by FreeStyle Optium Neo H in jugular (A) and ear (B) vein and by the reference method (Randox) in serum in ewes. Samples (*n* = 175) were collected from late gestation and postparturient ewes in the Aegean and Central Anatolian regions of Türkiye. The diagonal grey line (45°) is the identity line, while the dark line is the regression line. The blue area between dashed lines represents 95% confidence interval.

The presence of systematic and proportional bias was assessed by Passing–Bablok regression analysis (Figure 3). The analysis showed different slopes and intercepts between the serum BHBA concentrations measured by the reference method and the whole‐blood BHBA concentrations measured with FreeStyle Optium Neo H for the two veins. More specifically, for the jugular vein samples, the intercept and slope values were, respectively, −0.125 (95% CI: −0.148 to −0.078) and 1.250 (1.111 to 1.304), while for the ear vein samples, the intercept and slope values were, respectively, −0.050 (95% CI: −0.089 to −0.018) and 1.000 (0.909–1.111) (Table 2).

**TABLE 2:** Results of Passing–Bablok regression equations between reference method (Randox, in serum) and test device FreeStyle Optium Neo H (in whole‐blood from jugular and ear vein) for the analysis of beta‐hydroxybutyric acid (mmol/L) in ewes.

	Intercept (95% CI)	Slope (95% CI)
Jugular vein	−0.125 (−0.148 to −0.078)	1.250 (1.111 to 1.304)
Ear vein	−0.050 (−0.089 to −0.018)	1.000 (0.909 to 1.111)

The Se, Sp, positive predictive value (PPV) and negative predictive value (NPV) determined for the FreeStyle Optium Neo H device, using optimized thresholds based on the reference method (Randox) measurements, were 87.5 (95% CI: 47.3–99.7), 97.6 (95% CI: 94.0–99.3), 64 (95% CI: 39.1–82.7), and 99 (95% CI: 96.3–99.9), respectively, for jugular vein samples, and 87.5 (95% CI: 47.3–99.7), 91.0 (95% CI: 85.6–94.9), 32 (95% CI: 21.2–44.7), and 99 (95% CI: 96.0–99.9), respectively, for ear vein samples. As demonstrated by the AUC values, the whole‐blood BHBA thresholds of the FreeStyle Optium Neo H device in the jugular vein samples (0.896 [95% CI: 0.841–0.937]) and ear vein samples (0.887 [95% CI: 0.831–0.930]) successfully distinguished between the healthy and hyperketonaemic ewes (*p* < 0.001). Table 3 presents the AUC values and diagnostic test characteristics of the FreeStyle Optium Neo H device for the detection of hyperketonaemia in sheep with the use of optimized thresholds

**TABLE 3:** Diagnostic test characteristics of the FreeStyle Optium Neo H device for the detection of hyperketonaemia in sheep with the use of optimized thresholds obtained ROC analyzes based on the measurements of the reference method (Randox) in jugular and ear veins.

Site	Reference method thresholds (mmol/L)	Optimum threshold (mmol/L)	Sensitivity (%) (95% CI)	Specificity (%) (95% CI)	PPV (%) (95% CI)	NPV (%) (95% CI)	AUC (95% CI)
Jugular vein	≥0.8	≥0.8	87.5 (47.3–99.7)	97.6 (94.0–99.3)	64.0 (39.1–82.7)	99.0 (96.3–99.9)	0.896 (0.841–0.937)
Ear vein	≥0.8	≥0.6	87.5 (47.3–99.7)	91.0 (85.6–94.9)	32.0 (21.2–44.7)	99.0 (96.0–99.9)	0.887 (0.831–0.930)

*Note*: Samples were collected from late gestation and postparturient ewes (*n* = 175) in the Aegean and Central Anatolian regions of Türkiye.

Abbreviations: 95% CI, 95% confidence interval; AUC, area under the ROC curve; NPV, negative predictive value; PPV, positive predictive value; ROC, receiver operating characteristic.

When PT diagnosis was performed using the optimized thresholds, the Cohen's kappa coefficients (*κ*) calculated between the reference method (Randox) and FreeStyle Optium Neo H for the different blood collection sites ranged from *κ* = 0.43 to *κ* = 0.72 (Table 4).

**TABLE 4:** Cohen's kappa coefficients between the reference method (Randox) and FreeStyle Optium Neo H (FS) for the different blood collection sites, when optimized thresholds were used for diagnosing hyperketonaemi in sheep.

	Serum BHBA (Randox) vs. FS jugular vein BHBA	Randox BHBA vs. FS ear vein BHBA
Kappa (*κ*)	0.72	0.43
95% CI	0.49–0.95	0.21–0.65

Abbreviation: BHBA, beta‐hydroxybutyric acid.

## 4 Discussion

Monitoring BHBA concentrations is an important part of preventive veterinary medicine in sheep practice (Ji et al. 2023). Handheld ketone meters are widely used in the diagnosis of ketosis and pregnancy toxaemia in ruminants (Bach et al. 2016; Jansen et al. 2021; Panousis et al. 2018; Pichler, Damberger, Arnholdt, et al. 2014; Pichler, Damberger, Schwendenwein, et al. 2014). The present study evaluated the performance and diagnostic accuracy of the FreeStyle Optium Neo H device in determining whole‐blood BHBA concentrations at different blood collection sites in sheep. To the best of the authors’ knowledge, this is the first study to determine whole‐blood BHBA concentrations using FreeStyle Optium Neo H at two different sampling sites (jugular and ear veins) in sheep. In previous studies, the performance of handheld ketone meters in sheep was mostly evaluated using jugular blood samples. Only one study evaluated the accuracy of an electronic handheld meter (Precision Xceed) for diagnosing hyperketonaemia in ewes using both jugular and ear veins as sampling sites (Pichler, Damberger, Schwendenwein, et al. 2014). As with small animal medicine, it may be useful to use a minimally invasive blood sampling technique for monitoring hyperketonaemia in small ruminants. Accordingly, alongside the jugular vein, as the most commonly used sampling site in veterinary medicine, the authors selected the ear vein as another site that can be relatively easily sampled.

In general, high correlation coefficients have been reported between handheld ketone meters and the reference method in sheep (Araújo et al. 2020; Panousis et al. 2012; Pichler, Damberger, Schwendenwein, et al. 2014; Sjoberg and van Saun 2021; Turgut et al. 2024). Similar to a previous study (Tümer and Kılınç 2023), in which FreeStyle Optium Neo H was used to determine blood BHBA concentrations in sheep, a high correlation was found between the blood BHBA concentrations determined with FreeStyle Optium Neo H in samples from the jugular vein and serum BHBA concentrations determined by the reference method. Tümer and Kılıç (2023) also reported a high correlation (*r* = 0.882) between FreeStyle Optium Neo H and laboratory measurement. In this study, the Spearman's rho correlation coefficient between FreeStyle Optium Neo H and the reference method was 0.84. Consistent with a previous study using a minimally invasive sampling approach (Pichler, Damberger, Schwendenwein, et al. 2014), a high correlation was found between the blood BHBA concentrations measured with FreeStyle Optium Neo H in samples from the ear vein as an alternative to the jugular vein and serum BHBA concentrations measured by the reference method in samples from the jugular vein.

While the correlation coefficient can measure the strength of the relationship between FreeStyle Optium Neo H and the reference method, it cannot assess the agreement between them (Bland and Altman 1986). Therefore, Passing–Bablok regression analysis were used to evaluate the agreement between FreeStyle Optium Neo H and the reference method, and Bland–Altman plots were used to evaluate the bias of the two methods in this method comparison study

Passing–Bablok regression analysis is used in method comparison studies to identify systematic and proportional bias between two methods. This analysis has revealed constant and proportional errors in studies evaluating the usability of various point‐of‐care ketone meters, originally developed for humans, in cattle (Jansen et al. 2021; Yepes et al. 2018; Zakian et al. 2017) and sheep (Tümer and Kılınç 2023). In a previous study, using Passing–Bablok regression analysis, Tümer and Kılınç (2023) identified a positive constant (0.066) and positive proportional bias (1.242) for the FreeStyle Optium Neo H and a positive constant (0.625) and positive proportional bias (1.265) for TaiDoc in sheep. Yepes et al. (2018) reported a negative constant (−0.19, 95% CI: −0.23 to −0.14) and positive proportional bias (1.46, 95% CI: 1.41–1.50) for Precision Xtra in dairy cattle. Zakian et al. (2017) reported that Passing–Bablok regression analysis showed a linear relationship between Precision Xtra and the reference method. More specifically, the slope estimate of 1.03 (95% CI: 0.99–1.08) was not different from 1, while the intercept estimate of 0.03 mmol/L (95% CI: −0.01 to 0.06 mmol/) was not different from 0. Jones et al. (2021) also found no constant or proportional bias for Freestyle Precision Neo in dairy cows (intercept, −0.003, 95% CI: −0.07 to 0.06; slope, 1.009, 95% CI: 0.92–1.10). In this study, the Passing–Bablok regression analysis revealed a negative constant and positive proportional bias between the BHBA concentrations measured by FreeStyle Optium Neo H and the reference method in blood from the jugular vein. In contrast, a negative constant bias was detected between the blood BHBA concentrations measured with FreeStyle Optium Neo H in samples from the ear vein and the serum BHBA concentrations measured with the reference method in samples from the jugular vein (intercept did not include 0). However, proportional bias (95% CIs of the slope included 1 at both sites) was not detected. These findings indicate that FreeStyle Optium Neo H shows systematic and/or proportional bias compared to the reference method at two blood collection sites (jugular and ear veins), which means that FreeStyle Optium Neo H is not interchangeable for whole‐blood BHBA measurement in sheep. Cohen's kappa coefficient (*κ*) is a categorical agreement test and measures the reliability of two raters, taking into account the possibility that they may agree by chance (McHugh et al. 2012). The kappa coefficients in the present study showed that there was moderate‐to‐weak agreement between BHBA concentrations analyzed using the reference method and the FreeStyle Optium Neo H at both anatomical sites. This was in agreement with Passing–Bablok regression equation that showed systematic constant and proportional errors.

The Bland–Altman plot is widely used to assess the agreement between two measurement methods with limits of mean bias and agreement, and the 95% CI of agreement as the mean difference (±1.96 standard deviation). The 95% CI of agreement should include 95% of the difference between two measurement methods (Bland and Altman 1986). In our study, the Bland–Altman plots showed that the 95% CI of agreement included 95.4% (167/175) and 96.4% (169/175) of the difference between the results of the reference method and the FreeStyle Optium Neo H device for the samples from the jugular and ear veins. A strong agreement is supported when 95% of the differences between the means of the two methods are captured within the 95% CIs of the Bland–Altman plot. From a clinical perspective, the bias generated by Bland–Altman plots can be ignored because more than 95% of mean biases were captured by the 95% CI in Bland–Altman plots. Another ketone meter, CentriVet, overestimated blood BHBA concentrations in sheep with an average bias of 0.132 mmol/L (Turgut et al. 2024). On the other hand, Precision Xtra underestimated jugular and ear blood BHBA concentrations in sheep with a mean bias of 0.15 and 0.13 mmol/L, respectively (Pichler, Damberger, Schwendenwein, et al. 2014). Hornig et al. (2013) reported that Precision Xtra underestimated blood BHBA concentrations with an average bias of 0.06, whereas Ratanapob et al. (2019) reported that Precision Xtra showed no bias in measuring blood BHBA concentrations. Previous studies have reported that haematocrit affects blood BHBA concentrations measured by handheld ketone meters, with high haematocrit resulting in high blood BHBA concentration measurements (Megahed et al. 2017). The stability of analytes during the preservation process is also one of the preanalytical factors explaining these variations (Morris et al. 2002). Ambient temperature may affect the measurement of the FreeStyle Optium Neo H. According to the manufacturer, the performance of the handheld device used in this study was not affected by an ambient temperature ranging from +4 to +30. This study was conducted at different times of the year, including winter. The operator carried the device in the pocket before the analysis so that the FreeStyle Optium Neo H would not be affected by the ambient temperature. The difference between the results of the device and the reference method can be significantly influenced by the temperature of the tested sample (Iwersen et al. 2013). In this study, BHBA measurements were performed with the FreeStyle Optium Neo H immediately after blood samples were collected in order to analyze the sample before its temperature dropped. Moreover, an error message about temperature (E – 1 Error Message means that the temperature is too hot or too cold for the meter to work properly) was not received. After the test strip was placed into the device's test port, the blinking drop icon indicating the meter is ready to apply a sample to the test strip should appear in the display window. However only a few measurements were applied to the test strip too soon (before the blinking drop icon) by mistake (E – 5 Error Message means that blood was applied to the test strip too soon). These erroneous measurements were not included and repeated in this study. Apart from this, no error messages were received. This error message (E – 5) is not related meter error or performance. Therefore, the results this study were not limited by measurement errors.

Precision Xtra can identify sheep with hyperketonaemia (≥0.8 mmol/l) with 98.6% sensitivity and 98.2% specificity (Panousis et al. 2012), while Jones et al. (2018) reported that the Nova Vet Meter had 92.3% sensitivity and 94.5% specificity in identifying sheep with serum BHBA concentrations ≥0.8 mmol/L. These studies evaluated meter accuracy based on BHBA concentrations measured by a meter. Constant and proportional errors indicating non‐agreement with the reference method in the Passing–Bablok regression equation required us to adjust the Freestyle Neo H thresholds. Therefore, an ROC analysis was performed to determine the optimized thresholds for the device to detect hyperketonaemia in sheep. The ROC analysis identified thresholds of ≥0.8 and ≥ 0.6 mmol/L for the jugular and ear veins, respectively. The sensitivities and specificities of the optimized thresholds obtained in our study are consistent with the Se and Sp of the optimized thresholds (≥0.7 mmol/L for both sites) previously reported for the jugular and ear veins for Precision Xtra (Pichler, Damberger, Schwendenwein, et al. 2014). Pichler, Damberger, Schwendenwein, et al. (2014) reported that their optimized thresholds showed 82.6% sensitivity and 92.7% specificity for samples from the jugular vein and 81.2% sensitivity and 95.8% specificity for samples from the ear vein. However, when the AUC values ​​at the thresholds obtained in this study are taken into consideration, it is seen that FreeStyle Optium Neo H has good accuracy. These AUC values ​​show that the ability of FreeStyle Optium Neo H to distinguish healthy and hyperketonic sheep is good when using either blood sampling site (jugular and ear vein).

To protect sheep from PT, various preventive medical practices have been recommended, such as eliminating environmental stress, making ration adjustments, separating single and multiparous sheep or sheep carrying single and multiple foetuses, feeding sheep according to their physiological needs, and monitoring blood parameters throughout pregnancy (Ji et al. 2023). Handheld ketone meters have become important diagnostic tools for monitoring hyperketonaemia and early diagnosis of PT in sheep. In this study, the Bland–Altman plots showed that while the FreeStyle Optium Neo H device measured lower values than the reference method in samples from both blood collection sites, the measurement errors were acceptable if the 95% CI of the limit of agreement included at least 95% of the measurements. Regarding the circulating BHBA values, the FreeStyle Optium Neo H handheld ketone meter showed constant and proportional bias for the jugular vein yet good diagnostic performance in detecting sheep with PT or hyperketonaemia.

The most important limitation of the present study was the low prevalence of sheep with hyperketonaemia. PPV is affected by the prevalence of the disease in the flock. The relatively low PPV level seems to be related to the low prevalence of hyperketonaemia in this study. Low PPV results in unnecessary treatment of animals that the test classifies as diseased but are actually healthy. High NPV values ​​obtained at both measurement sites in this study reduce the probability of treatment due to misclassification. Another limitation of this study is missing the intra‐ and inter‐assay CVs at moderate and high BHBA concentrations. Calculating intra‐ and inter‐assay CVs ​​only at low BHBA concentrations resulted in intra‐ and inter‐assay CV values ​​of 0. Hence, the total error was not calculated. However, the Frestyle Optium Neo H showed 6.25% and 8.94% intra‐assay CVs at moderate and high BHBA concentrations, respectively (Tümer and Kılıç 2023).

In conclusion, despite the constant and proportional errors in the Passing–Bablok regression analysis, the acceptable negative low bias of FreeStyle Optium Neo H indicated by the Bland–Altman plots of agreement indicates that this device can provide rapid on‐site application for both jugular and ear vein blood sampling to produce reliable diagnosis of hyperketonaemia in ewes.

## Author Contributions

**Mehmet Akköse**: conceptualization, methodology, formal analysis, investigation, writing—original draft, writing–review and editing, supervision, project administration, funding acquisition. **Ufuk Kaya**: conceptualization, methodology, formal analysis, writing–original draft, writing–review and editing. **Murat Onur Yazlık**: conceptualization, investigation, writing–original draft, writing–review and editing. **Murat Göcen**: conceptualization, methodology, investigation, writing–original draft, writing–review and editing. **Mehmet Rıfat Vural**: conceptualization, methodology, writing–original draft, writing–review and editing.

## Conflicts of Interest

The authors declare no conflicts of interest.

## Peer Review

The peer review history for this article is available at https://www.webofscience.com/api/gateway/wos/peer‐review/10.1002/vms3.70592.

## Supporting information




**Supporting File 1**: vms370592‐sup‐SuppMat.xlsx.

## Data Availability

The datasets generated during and/or analyzed during the current study are available from the corresponding author on reasonable request.
